# Nitric Oxide-Releasing Microparticles: A Novel Treatment for Onychomycosis

**DOI:** 10.1021/acs.molpharmaceut.5c00613

**Published:** 2025-08-21

**Authors:** Alessandro F. Valdez, Jhon Jhamilton Artunduaga Bonilla, Daniel Zamith-Miranda, Bruna Montalvão, Éva Veres, Sirida Youngchim, Clay Tucker, Andrew Draganski, Attila Gácser, Leonardo Nimrichter, Joshua D. Nosanchuk

**Affiliations:** † Laboratório de Glicobiologia de Eucariotos, Departamento de Microbiologia Geral, Instituto de Microbiologia Paulo de Góes, 341406 Universidade Federal do Rio de Janeiro, Rio de Janeiro, Rio de Janeiro 21941-902, Brazil; ‡ 2006 Albert Einstein College of Medicine, Departments of Medicine (Division of Infectious Diseases) and Microbiology and Immunology, Bronx, New York 10461, United States; § Department of Biotechnology and Microbiology, University of Szeged, Szeged 6726, Hungary; ∥ Department of Microbiology, Faculty of Medicine, 37686 Chiang Mai University, Chiang Mai 50200, Thailand; ⊥ 716565 Zylö Therapeutics, Greenville, South Carolina 29615, United States; # Rede Micologia RJ, Carlos Chagas Filho Foundation for Research Support of the State of Rio de Janeiro (FAPERJ), Rio de Janeiro, Rio de Janeiro 20020-000, Brazil

**Keywords:** onychomycosis, T. rubrum, T. mentagrophytes, C. albicans, microparticles, nitric oxide

## Abstract

Onychomycosis is one of the most prevalent fungal infections worldwide, resulting in negative effects on general quality of life. Management of the disease encounters a series of obstacles, and antifungal treatment has a low success rate. We aimed to develop and evaluate a novel formulation of NO-releasing microparticles (SNO-MPs) against some of the most common causative agents of onychomycosis: *Trichophyton mentagrophytes*, *Trichophyton rubrum*, *Candida albicans*, and *Aspergillus flavus*. SNO-MP susceptibility testing was performed *in vitro,* and MICs and MFCs were established for multiple strains. SNO-MP was also tested *ex vivo* in human nail fragments, with biofilm formation and biofilm disruption assessed using scanning electron microscopy and colony-forming unit counting. The particles demonstrated the ability to destroy biofilms formed by either *Trichophyton* sp. or *C. albicans*. SNO-MP was not effective against *A. flavus*, which appears to have a nitrosative resistance mechanism that has not yet been elucidated. Cytotoxicity was assessed against human dermal fibroblasts and keratinocytes. SNO-MP demonstrated a manageable degree of cytotoxicity when tested against human dermal fibroblasts and keratinocytes. Our findings highlight that SNO-MP represents a potentially potent alternative for the treatment of onychomycosis due to *Trichophyton* and *Candida*.

## Graphical abstract

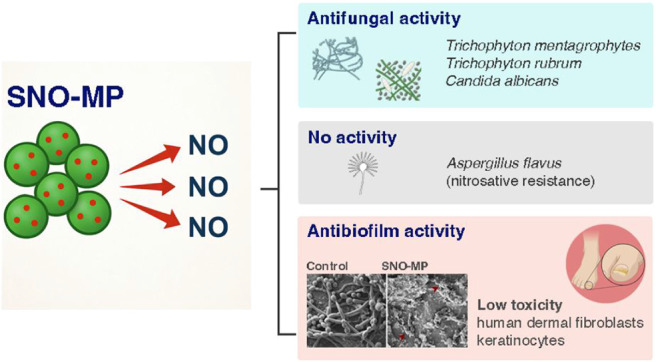

## 1 Introduction

Onychomycosis is a fungal nail infection that affects approximately 10% of the global population across all cultures and ethnicities, and it is the most prevalent nail disease worldwide.^1–3^ The most frequent causative agent of onychomycosis is *Trichophyton rubrum* followed by *Trichophyton mentagrophytes*, *Epidermophyton floccosum*, *Microsporum* spp., and other dermatophytes,^1^ but the disease is also caused by *Aspergillus flavus* and yeast-like fungi such as *Candida albicans* and *Candida parapsilosis*.^4,5^ Despite being a superficial disease, onychomycosis is a serious global health problem, accounting for 30% of all superficial fungal infections, 90% of all toe-nail infections, and 50% of all fingernail infections.^2,6,7^ Onychomycosis causes pain to the affected digit and disfiguration of the nail, usually discoloration and thickening of the nail plate. The disease is more frequent on toenails due to slower nail growth rate, frequent confinement in dark, humid environments, and overall lower vascularization of the peripheral tissue.^1–3^ Although onychomycosis has a low morbidity, the disease is associated with major negative psychological effects that diminish patients’ overall well-being and quality of life.^8^

Treatment for onychomycosis relies on antifungal drugs administered either topically or systemically, with both treatment regimens being protracted (e.g., months to years) and expensive, factors that affect patient adherence rates.^9,10^ Furthermore, the management of onychomycosis encounters intrinsic obstacles: topical antifungals and their active molecules face the barrier of the nail plate, which is highly keratinized and therefore difficult to penetrate; systemic antifungals have limited access to nails due to low vascularization; and the main antifungals carry hepatotoxicity risks.^3,9,10^ Moreover, the antifungals utilized have low bioavailability and lack selectivity, and there are increasing reports of antifungal resistance.^11,12^

In this context, micro- and nano-drug delivery systems (MiNaDDS) have become an interesting alternative by providing a platform to optimize molecules with proven antifungal action, while potentially countering many of the limitations already described.^11–13^ When applied topically, MiNaDDS offer enhanced cutaneous penetration and sustained release of the entrapped, encapsulated, or bound drug to the targeted tissue.^13–15^ To combat onychomycosis, our group developed a silane hydrogel platform to topically deliver nitric oxide (NO). Beyond its effects on skin homeostasis, NO is an immune response modulator with proven antibacterial and antifungal activity.^16–18^ In fact, previous studies from our group demonstrated that early formulations of this NO-releasing microparticle were able to inhibit fungal growth in multiple experimental settings,^18–21^ including investigations in the context of onychomycosis, with promising results against *T. rubrum* planktonic cells and inhibition of biofilm formation *in vitro*.^22,23^

In this study, our goal was to generate a novel microparticle (SNO-MP) that releases *S*-nitrosothiol (SNO), inducing a sustained release of NO, for use in the treatment of onychomycosis. We found that the SNO-MP had potent efficacy, as determined by minimal inhibitory concentration (MIC) and minimal fungicidal concentration (MFC) testing, against multiple strains of dermatophytes (*T. rubrum* and *T. mentagrophytes*) and *C. albicans*, although *A. flavus* strains displayed MICs >20 mg/mL. Direct topical application to human nails in an *ex vivo* model of infection demonstrated potent fungicidal effects, with total or nearly total destruction of the *Trichophyton* and *Candida* structures present in the nail fragments. Cytotoxicity assays performed with human immortalized cells demonstrated a manageable degree of cytotoxicity. Taken together, our data strongly support the further development of SNO-MP for the treatment of onychomycosis caused by dermatophytes and *Candida*.

## 2 Materials and Methods

### 2.1 Fungal Cultures and Strains

*C. albicans* strains were cultured in Sabouraud media (Difco #238230). *T. rubrum* and *T. mentagrophytes* and *A. flavus* strains were cultured in potato dextrose agar (PDA – Difco #213400). Clinical strains were obtained from our hospital (Montefiore Medical Center) and through donations by multiple investigators (Table S1).

### 2.2 SNO-MP Synthesis and Nitrosation

SNO-MPs were synthesized as described in Tar et al., 2022.^24^ Briefly, the process consists of (i) acid-catalyzed hydrolysis of two silicate precursors, tetraethylorthosilicate (TEOS) and mercaptopropyltrimethoxysilicate (MPTS); (ii) co-condensation of hydrolyzed TEOS and MPTS to form a highly porous thiolated sol–gel monolith; (iii) a series of proprietary steps to remove any unreacted monomer and nanosized particulate matter; and (iv) a drying step to evaporate residual water and ethanol.

For use, the SNO-MP required activation, which was achieved by suspending unnitrosated microparticles in cold methanol, after which cold HCl was added and mixed by vortexing. Nitrosation was achieved by adding cold sodium nitrite to the unnitrosated SNO-MP, upon which the suspension turned pink immediately, indicating the presence of NO. After the neutralization of the suspension with NaOH, nitrosated SNO-MPs were spun down, the supernatant was discarded, and the microparticles were suspended in cold RPMI/MOPS to achieve the desired particle concentration. NO kinetics were measured as described previously.^25^ In brief, measured amounts (mg) of SNO-MP particles were added to 50 mM phosphate buffer at pH 7.4 in a vessel which was thoroughly purged of oxygen with high-purity nitrogen gas. The nitrogen gas was continuously sparged through the vessel so that nitric oxide released from the particles was carried to a reaction chamber and chemiluminescent sensor of a NO analyzer (Sievers NO Analyzer 280i; GE Analytical Instruments, Boulder, CO). NO flux peaked at 1.8 nmol/mg/min within the first 10 min followed by a logarithmic decay and then was sustained at 0.4 nmol/mg/min for approximately 64 h. The NO storage capacity of SNO-MP has been determined to be approximately 2.5 μmol/mg. SNO-MP’s use has been described in several prior publications.^24–27^

### 2.3 Sustained NO Release by SNO-MP

The mass of NO released per milligram of SNO-MP was determined under conditions identical to those of the susceptibility tests and cytotoxicity assays (e.g., growth medium composition and pH, incubation time and temperature, and rate and amplitude of vessel shaking). SNO-MPs were dispersed in media (RPMI/MOPS) at various concentrations (5, 10, and 20 mg/mL) and placed in the wells of a 96-well plate. The plate was then covered and placed on an orbital shaker in an incubator protected from light. At different times, the 96-well plate was removed, and the entire contents of a single well were extracted to be quantified for NO. Quantification was by a NO analyzer in a similar fashion as described above. Briefly, the contents were added to a purge vessel containing pH 7.4 phosphate buffer that was continuously sparged with high-purity nitrogen and carried to the NO analyzer. Liberation of NO from SNO-MP was accelerated by shining a bright, broad-spectrum white light on the purge vessel. Total NO released was determined by summing the area under the curve.

### 2.4 *C. albicans* SNO-MP Microplate Susceptibility Test

Working solutions of activated SNO-MP were prepared in RPMI 1640 buffered with MOPS (MOPS: 0.165 mol/L, pH 7), and 2-fold concentrations were plated onto 96-well round-bottom plates in a volume of 100 μL. *C. albicans* suspensions were prepared by serially diluting 24 h cultures from each strain to match a 0.5 McFarland standard at 530 nm wavelength with the help of a spectrophotometer, in accordance with the CLSI M27 protocol.^28^ Finally, 100 μL of the fungal suspensions was then plated to achieve a final volume of 200 μL/well, adjusting SNO-MP to final concentrations of 20, 10, 5, and 1 mg/mL and fungal suspensions to final concentrations of 2.5 × 10^2^ to 1.25 × 10^3^ cells/mL in RPMI/MOPS.

After inoculation, the plates were incubated at 37 °C for 48 h under agitation at 150 rpm. Following incubation, metabolic activity was measured using XTT (2,3-bis­(2-methoxy-4-nitro-5-sulfophenyl)-2*H*-tetrazolium-5-carboxanilide/Thermo Fisher, X6493). Briefly, a solution of XTT (0.5 mg/mL) and menadione (10 mM, acting as an electron coupling agent) was prepared and incubated at 37 °C for 2 h. Color development was monitored by absorbance reading at 490 nm, and MICs were labeled as the first group that had metabolic activity dampened by at least 90% in comparison to the control group (no treatment). XTT was selected for use in these experiments because SNO-MP turbidity impaired visual or spectrophotometric MIC readings for several higher concentrations tested. The experiments were performed with three replicates per concentration in each experiment, and three independent experiments were conducted.

### 2.5 Dermatophytes and *Aspergillus* SNO-MP Microplate Susceptibility Test

Working solutions of activated SNO-MP were freshly prepared as described above. Dermatophyte (*T. rubrum* and *T. mentagrophytes*) and *A. flavus* conidia suspensions were prepared in accordance with the CLSI M38 protocol^29^ and diluted to a final concentration of 1 × 10^3^ to 3 × 10^3^ CFU/mL and 0.4 × 10^4^ to 5 × 10^4^ CFU/mL, respectively, in RPMI/MOPS, to a final volume of 200 μL, adjusting SNO-MP to a final concentration of 20, 10, 5, and 1 mg/mL.

After inoculation, *A. flavus* plates were incubated for 48 h at 30 °C, and *Trichophyton* sp. plates were incubated for 72 h at 30 °C in accordance with CLSI M38, under agitation at 150 rpm. Following incubation, MICs were determined by visual analysis. MICs were defined as the total absence of growth (score of 0). The experiments were performed with three replicates per concentration in a total of three independent experiments.

### 2.6 Minimal Fungicidal Concentrations (MFCs)

Minimal fungicidal concentrations were determined by subculturing 10 μL from wells corresponding to MIC and above concentrations onto PDA agar plates. Plates were incubated for the same duration and temperature utilized in each MIC test, and then, colonies were counted. MFC was defined as the lowest concentration that yielded no growth at all or fewer than three colonies.^30^ The experiments were performed with three replicates per concentration in three independent experiments.

### 2.7 SNO-MP Activity in an *Ex Vivo* Nail Infection

The SNO-MP activity against biofilms formed in human nails was determined by using scanning electron microscopy (SEM) and quantified by CFUs. Nail fragments were collected from healthy individuals under the approval of the Universidade Estadual de Maringá Ethical Committee on Human Experimentation (#31,702,520.40000.0104) and were kindly donated by Dr. Melyssa Negri for this study. The fragments were sterilized by immersion in 70% ethanol for at least 3 h, washed three times with PBS, and transferred to tubes containing 200 μL of RPMI/1% PenStrep with 1 × 10^6^ CFU/mL from a single fungal species. *C. albicans* cells were incubated at 37 °C for 48 h, and *Trichophyton* sp. conidia were incubated at 30 °C for 72 h. Afterward, nail fragments were washed three times with PBS to remove nonadherent cells. Biofilm formation was confirmed using an optical microscope (Olympus CX-31), and then, 500 μL of SNO-MP (10 or 20 mg/mL) previously diluted in RPMI/MOPS was added. After incubation under the conditions utilized for biofilm formation, the nail samples were washed with PBS, and the tissue and fungal cells were fixed with 2.5% glutaraldehyde and 4% formaldehyde (in 0.1 M cacodylate buffer, pH 7.2) for 1 h at room temperature. The samples were then washed with cacodylate buffer, dehydrated using an ethanol gradient, coated with a 20 nm layer of gold, and visualized using an SEM (ZEISS EVO MA 10, Germany) operated at 10 kV. Terbinafine (TRB – at 32 and 64 μg/mL) and amphotericin B (AmB – at 1 μg/mL) were used as treatment controls for dermatophytes and *C. albicans*, respectively. Untreated infected nails were used as an additional control.

To determine CFUs, samples from the control and treated nail fragments were collected and weighed prior to the fixation stage. The samples were suspended in 200 μL of RPMI 1640/1% PenStrep, subjected to sonication for 1 min, and vortexed. Aliquots of 20 μL were plated onto Sabouraud plates. Following incubation at the respective temperatures for 2–3 days, CFU determinations were made. Two independent experiments were conducted.

### 2.8 Cytotoxicity

*In vitro* cytotoxicity experiments were performed with human dermal fibroblasts (HFb, ATCC CRL-PCS-201-401) and human keratinocytes (HaCat). Both cell lines play critical roles in nail bed regeneration. Keratinocytes are the main epithelial cell type present and function to maintain and reestablish the epithelial surface. Fibroblasts are present in and around the nail, especially as onychofibroblasts, and are responsible for rebuilding the supportive dermal environment.^31,32^ The chosen cells were both cultivated in RPMI supplemented with 10% fetal bovine serum at 37 °C with 5% CO_2_ until cell confluence was obtained. A total of 1 × 10^5^ and 1 × 10^4^ cells/well were plated in 96-well plates, respectively, where they were treated with SNO-MP over a dilution range of 1–100× MFC90 for 72 h. Untreated cells served as the control. After treatment, cell viability was measured via XTT assays, as previously described in Topic 2.4, and CC_50_ was determined as the concentration that reduced cell viability by 50% when compared to untreated controls. Experiments were performed with two replicates per concentration for a total of three independent experiments.

## 3 Results

### 3.1 SNO-MP NO Release

Nitric oxide release over time was measured under a range of different experimental conditions, encompassing the conditions detailed in the CLSI protocols. The results enabled the correlation of SNO-MP weight with NO release at different incubation times and temperatures (Table 1). Conditions 2, 3, and 4 were used for experiments with *C. albicans*, *A. flavus*, and *Trichophyton* sp., respectively.

**Table 1:** Total NO Released under Different Experimental Conditions

Incubation conditions				Total NO released (μg/mL) at:					
	Temperature (°C)	Time (h)	Species	5 mg/mL	SD	10 mg/mL	SD	20 mg/mL	SD
1	37	8	*C. albicans*	383.5	28.3	689.8	44.1	1361.3	113.2
2	37	24	*C. albicans*	451.2	7.7	846.1	82.9	1758.2	43.3
3	30	48	*A. flavus*	448.7	14.2	1007.0	22.8	1849.0	134.6
4	30	72	*Trichophyton* sp.	493.5	24.0	1074.1	5.0	1994.9	118.3
5	30	96	*Trichophyton* sp.	523.4	15.5	1079.8	25.5	2126.7	71.9

### 3.2 SNO-MP Minimal Inhibitory and Fungicidal Concentrations

MICs and MFCs of SNO-MP were determined for a total of 26 *C. albicans* strains (Table 2). The SNO-MP MIC was determined to be 10 mg/mL, with the exception of 4 strains—90028, Sc5314, AV18, and AV25—that had MICs of 5 mg/mL. Notably, MFCs for most *C. albicans* strains were the same as MICs, with the exception of strains Sc5314, AV20, and AV25, which showed a one-fold increase. MIC90 and MFC90, defined as the lowest concentrations with the corresponding effect in at least 90% of the strains tested, were determined to be 10 mg/mL of SNO-MP.

**Table 2:** *Candida albicans* MICs and MFCs

Strain	MIC (mg/mL)	MFC (mg/mL)	Strain	MIC (mg/mL)	MFC (mg/mL)	Strain	MIC (mg/mL)	MFC (mg/mL)
90028*	5	5	AV09	10	10	AV18	5	5
Sc5314*	5	10	AV10	10	10	AV19	10	10
AV01	10	10	AV11	10	10	AV20	10	20
AV02	10	10	AV12	10	10	AV22	10	10
AV03	10	10	AV13	10	10	AV23	10	10
AV05	10	10	AV14	10	10	AV24	10	10
AV06	10	10	AV15	10	10	AV25	5	10
AV07	10	10	AV16	10	10	AV26	10	10
AV08	10	10	AV17	10	10			

For dermatophyte molds, a total of 27 strains were analyzed, with 14 being *T. rubrum* (Table 3) and 13 *T. mentagrophytes* (Table 4). SNO-MP MICs were 10 mg/mL for 8 of the 14 *T. rubrum* and 9 of the 13 *T. mentagrophytes* strains, with the others having an MIC of 20 mg/mL. For *T. rubrum*, the MFCs were the same as the MICs, with the exception of BR1A and DI23118, where the MICs were 10 mg/mL and the MFCs 20 mg/mL. Five strains of *T. mentagrophytes* went from MICs of 10 mg/mL to MFCs of 20 mg/mL, and the rest had equal MICs and MFCs. In total, 17 of the 27 dermatophyte strains had MFCs of 20 mg/mL. MIC90 and MFC90 were determined at 20 mg/mL of SNO-MP.

**Table 3:** *T. rubrum* MICs and MFCs

Strain	MIC (mg/mL)	MFC (mg/mL)	Strain	MIC (mg/mL)	MFC (mg/mL)	Strain	MIC (mg/mL)	MFC (mg/mL)
BR1A	10	20	DI23110	10	10	DI23118	10	20
FM1064	20	20	DI23112	20	20	DI23119	10	10
R1	10	10	DI23113	10	10	PMN01	20	20
AV01	10	10	DI23114	20	20	PMN02	20	20
CFP900	10	10	DI23116	20	20			

**Table 4:** *T. mentagrophytes* MICs and MFCs

Strain	MIC (mg/mL)	MFC (mg/mL)	Strain	MIC (mg/mL)	MFC (mg/mL)	Strain	MIC (mg/mL)	MFC (mg/mL)
53062	10	10	DI23103	20	20	PMN01	20	20
AV01	10	20	DI23104	10	10	PMN02	20	20
AV02	10	20	DI21100	10	20	PMN03	10	20
R1	10	10	DI23107	10	10			
FM1090	10	20	DI23108	20	20			

For nondermatophyte molds, a total of 15 strains of *A. flavus* were tested. Unfortunately, the SNO-MP formulation did not inhibit any *A. flavus* strain at concentrations up to 20 mg/mL SNO-MP (data not shown).

### 3.3 SNO-MP Activity in *Ex Vivo* Human Nail Infections

SNO-MP activity against fungal cells was tested in an *ex vivo* infection setting. Human nail fragments were incubated with individual strains of *C. albicans*, *T. rubrum*, and *T. mentagrophytes*. SEM demonstrated that SNO-MP at either 10 or 20 mg/mL concentrations had significant antibiofilm activity, killing the fungi infecting the nail fragments while also coating the nail fragments with SNO-MP. SNO-MP coating on control nail fragments can be observed in Figure S1, and representative photographs obtained by SEM for *C. albicans*, *T. rubrum*, and *T. mentagrophytes* are shown (Figures 1 and 2). For *C. albicans*, filamentous and yeast forms are diffusely present in untreated infected nails. In treated infected nails, the fungal cells are mostly absent, with rare cells remaining that are collapsed, shortened, or fractured in both the 10 and 20 mg/mL concentrations (Figure 1).

**Figure 1:**
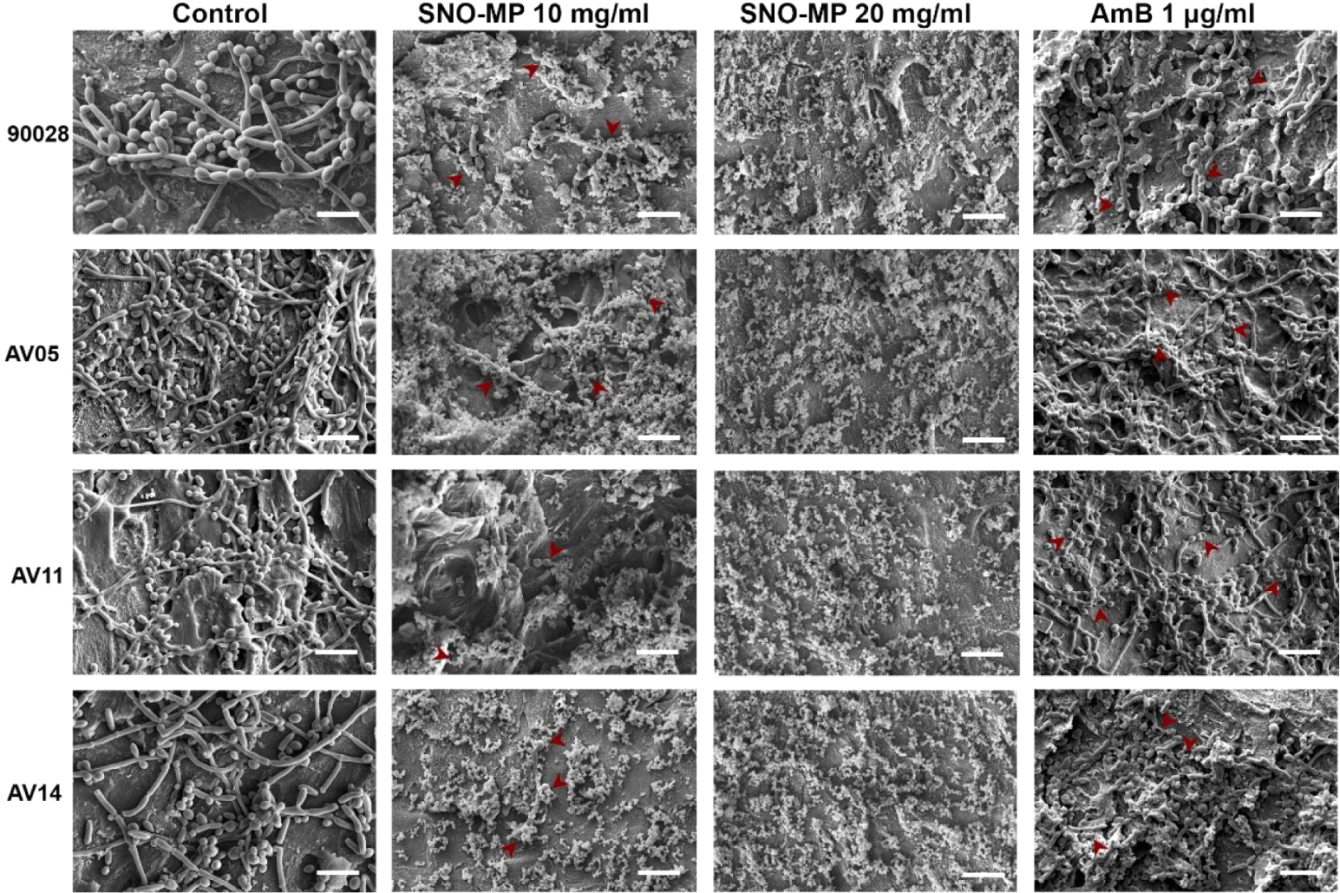
*Ex vivo* efficacy of SNO-MP against *C. albicans*: nail fragments were incubated with *C. albicans* yeasts for 24 h at 37 °C. The fragments were then incubated with SNO-MP (10 or 20 mg/mL) or 1 μg/mL of AmB. Untreated infected fragments were used as controls. The micrographs show that the control group had yeasts abundantly and uniformly occupying the entire nail surface. Treatment with SNO-MP at either concentration resulted in the destruction of fungal structures, with the few remaining yeasts showing signs of collapse and cellular stress (red arrows), similar to the treatment with AmB. Scale bar = 15 nm. Representative images were obtained at a magnification of 3000×.

**Figure 2:**
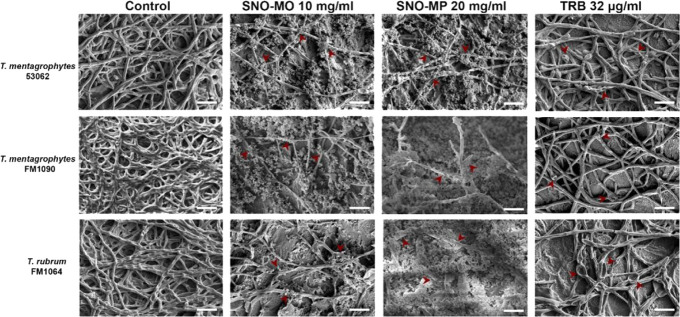
*Ex vivo* efficacy of SNO-MP against *Trichophyton* sp.: nail fragments were incubated with *Trichophyton* sp. for 72 h at 37 °C. The fragments were then incubated with SNO-MP (10 or 20 mg/mL) or 32 μg/mL of TRB. Untreated infected fragments were used as controls. The micrographs show that the control group has hyphae abundantly and uniformly occupying the entire nail surface. Treatment with SNO-MP at either concentration resulted in the destruction of the fungal structures, with the few remaining hyphae showing signs of collapse and cellular stress (red arrows), similar to treatment with TRB. Scale bar: 15 nm. Representative images at a magnification of 3000×.

Similarly, *Trichophyton* sp. extensively colonized the nail plate in untreated infected nails. Nails treated with either 10 or 20 mg/mL SNO-MP showed significant biofilm destruction, with the remaining hyphae demonstrating an overall flattened morphology and multiple collapse points (Figure 2). In both *C. albicans* and dermatophyte strains, SNO-MP treatment resulted in a disruption of biofilm and cell morphology similar to those of their respective antifungal controls, AmB (1 μg/mL) and TRB (32 μg/mL). The effects of SNO-MP on both *C. albicans* and *T. mentagrophytes* are shown in greater detail in Figures 2 and S3, respectively.

These results were corroborated by CFU measurements from infected nail fragments that showed a dose-dependent effect against the *C. albicans* (Figure 3) and dermatophyte strains (Figure 4) tested. Notably, the SNO-MP activity was similar to amphotericin B (1 μg/mL: yeast control) and both terbinafine concentrations (32 and 64 μg/mL: dermatophyte controls) in both the SEM and CFU experiments. The TRB 64 μg/mL control was added only to the CFU experiment to provide a better comparison when SNO-MP was more effective than TRB 32 μg/mL.

**Figure 3:**
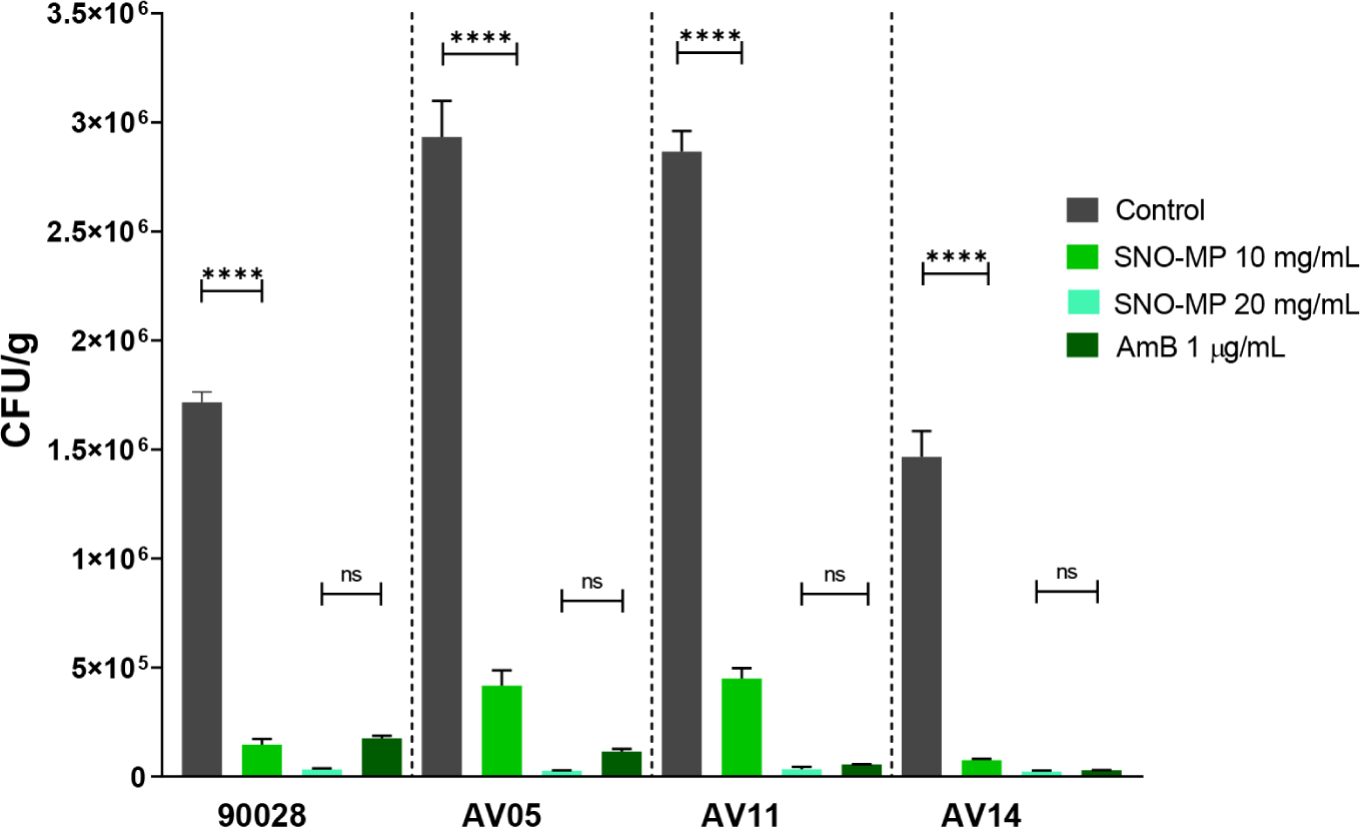
CFU analysis of nails infected with *C. albicans*: CFU analysis demonstrates that treatment with SNO-MP was effective against all strains at 10 and 20 mg/mL concentrations, with the 20 mg/mL concentration having a similar effect to AmB 1 μg/mL. Statistical analysis was performed using ordinary one-way ANOVA with multiple comparisons (p**** < 0.0001).

**Figure 4:**
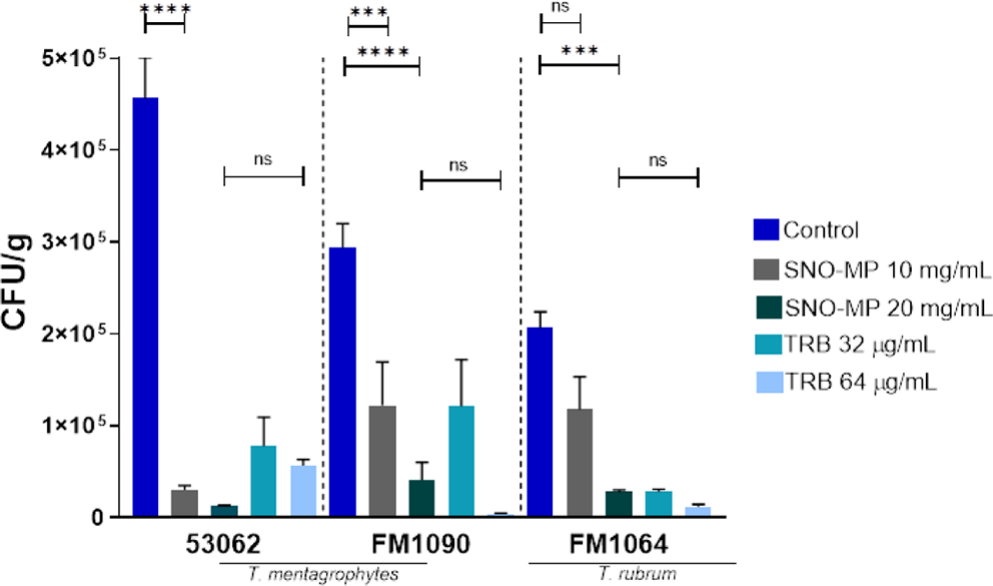
CFU analysis of nails infected with *Trichophyton* sp.: CFU analysis demonstrates that treatment with SNO-MP was effective on all strains at 10 and 20 mg/mL concentrations, except for strain FM1064, for which the 10 mg/mL concentration did not show a significant difference compared to the untreated group. The 20 mg/mL concentration was effective against all strains, with an effect comparable to TRB 64 μg/mL. Statistical analysis was performed using ordinary one-way ANOVA with multiple comparisons (*p* *** < 0.0005, **** < 0.0001).

### 3.4 SNO-MP *In Vitro* Cytotoxicity

*In vitro* cytotoxicity was determined in human dermal fibroblasts (HFb) and human keratinocytes (HaCat). Concentrations of SNO-MP ranging from 0.15 to 20 mg/mL were incubated with cells, and XTT readings were compared to the control condition (no treatment). CC_50_ curves demonstrated a CC_50_ of 9.5 mg/mL for HaCat cells (Figure 5A) and 1.21 mg/mL for HFb cells (Figure 5B).

**Figure 5:**
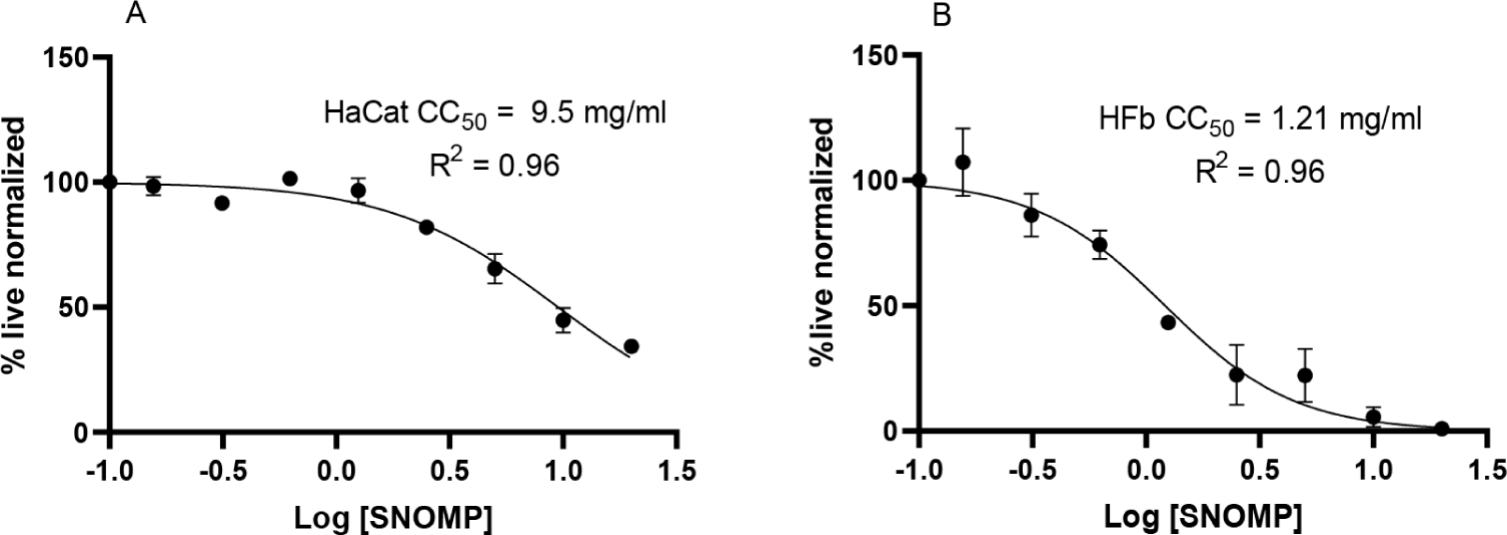
CC50 curves of immortalized human keratinocytes (HaCat – A) and dermal fibroblasts (HFb – B). After incubation with SNO-MP in different concentrations (20 to 0.15 mg/mL), normalized XTT readings were compared to the control condition (no treatment) and plotted in a nonlinear fit against log [SNO-MP] using the GraphPad Prism software version 9.2.0. The resulting CC_50_ was 9.5 mg/mL SNO-MP for HaCat (A) and 1.21 mg/mL SNO-MP for HFb (B). Both nonlinear regressions had an *R* ^2^ of 0.96.

## 4 Discussion

Onychomycosis is a highly prevalent and traumatizing nail infection that poses a series of therapeutic challenges, including cost, treatment duration, patient compliance, drug bioavailability, and antifungal resistance. Micro- and nano-drug delivery systems offer an alternative to address several of these limitations. In this context, our group developed and tested a novel formulation of NO-releasing microparticles for onychomycosis treatment.

The results demonstrate that our SNO-MP effectively released NO in a dose-dependent manner with little to no variation between 30 and 37 °C over time (Table 1). These observations not only allowed us to correlate microparticle concentration with NO inhibitory activity and fungicidal concentrations, but also provided insights into the effects that time and temperature might have on treatment.

NO-releasing particles have their antimicrobial effects well described in the literature, including recent accounts of antifungal effects.^18–21,27,33^ However, in the context of onychomycosis, investigations are scarce, and studies have been conducted with a limited number of species and strains.^22,23,34,35^ In fact, our group previously described the potential of an earlier formulation of a NO-releasing microparticle against two *T. rubrum* strains, demonstrating its efficacy against planktonic cells and biofilms *in vitro*.^22,23^

With this in mind, we tested SNO-MP against a panel of diverse strains to determine the MICs and MFCs across different species that cause onychomycosis. Analyses of 26 *C. albicans* strains tested in this study, including 2 reference strains, revealed an MIC90 and MFC90 of 10 mg/mL SNO-MP or 591 μg/mL of NO, confirming what was observed in earlier studies^18,19^ (Table 2). Data from the 15 *T. rubrum* and 13 *T. mentagrophytes* strains revealed MIC90 and MFC90 values for both species of 20 mg/mL SNO-MP or 1393 μg/mL NO (Tables 3 and 4). However, the average MIC for *Trichophyton* sp. was lower than the MFC, suggesting that SNO-MP has a fungistatic effect in dermatophytes at 10 mg/mL, while the same concentration was fungicidal for the *C. albicans* strains.

Although SNO-MP had excellent effects against *Candida* and dermatophytes, the particle had no inhibitory or fungicidal effects against *A. flavus* at the concentrations tested. To our knowledge, there are no accounts of NO resistance mechanisms described for *A. flavus*. However, NO-tolerant proteins responsible for reducing environmental NO and nitrite levels have been described in *A. nidulans*.^36^ Notably, the efficacy of small-molecule NO donors against *A. fumigatus* was recently described.^33^ Taken together, our results and literature accounts suggest that NO efficacy and resistance mechanisms may be species-specific, and further experimentation will be necessary to elucidate *A. flavus* nitrosative stress detoxification.

Human nail fragments were utilized to visualize SNO-MP effects in an onychomycosis-specific *ex vivo* assay. Scanning electron micrographs show that both *C. albicans* and *Trichophyton* sp. successfully colonize and form a biofilm on the nails *ex vivo*. Nail treatment was then performed, and micrographs suggest that SNO-MP is highly effective at destroying biofilm and protecting the nail surface. Figure 1 demonstrates that both 10 and 20 mg/mL SNO-MP destroy *C. albicans* biofilms in each of the 4 strains tested, and the very few remaining yeasts encountered present signs of heavy cellular stress, such as deformed cell walls and budding impairment (indicated in the images by red arrows). These observations were confirmed by CFU counts performed with the infected nail fragments. Treatment with 10 mg/mL SNO-MP demonstrated an average 5-fold CFU reduction in *Candida* yeasts, while 20 mg/mL SNO-MP has an antifungal activity comparable to 1 μg/mL AmB (Figure 3). *C. albicans*’ ability to form biofilms is an important virulence factor for establishing disease on tissue, including nails.^37^ In this context, it is worth highlighting that SNO-MP could also be explored as an alternative tool for the eradication of biofilms. Notably, we previously demonstrated the ability of an earlier formulation of NO particles to prevent *C. albicans* biofilms in central venous catheters placed in rats.^19^ Similarly, micrographs of *Trichophyton* sp.-infected nail plates treated with SNO-MP corroborated the overall treatment efficacy (Figure 2). In contrast to what was observed for *C. albicans*, the sub-MIC90 concentration (10 mg/mL) was not as efficient in destroying biofilm, though it was comparable to the effects of TRB at 32 μg/mL. However, treatment with 20 mg/mL SNO-MP proved to be potently effective against dermatophyte biofilms, and its effects were comparable to TRB at 64 μg/mL (Figure 4). CFU counts revealed a corresponding pattern to the imaging. The only treatment that showed no statistical significance was the 10 mg/mL SNO-MP for strain FM1064, to which this concentration was sub-MIC. Taken together, when compared to NO-releasing formulations previously used by our group, our results demonstrated an increase in antifungal capability against *Trichophyton* sp. In fact, our current formulation was capable of disrupting biofilms formed by dermatophytes with 10 mg/mL SNO-MP and above, while previous formulations had similar results with 40 mg/mL.^23^

Our cytotoxicity results showed that HaCat cells displayed a CC_50_ of 9.5 mg/mL, while the HFb CC_50_ was significantly lower at 1.21 mg/mL (Figure 5). Given that the SNO-MP is intended for use directly on nails with limited interactions with either epithelial cells or fibroblasts, as well as the fact that skin has a rapid turnover of cells, these values are well within manageable ranges. Notably, the same formulation recently demonstrated no hemolytic activity,^38^ and earlier iterations of our nitric oxide particles have been administered without demonstrated toxicity in several animal models, including systemic administration to counter lipopolysaccharide (LPS)-induced endotoxemia in a mouse model^39^ or hemorrhagic shock in mice^40^ where the nitric oxide-releasing particles were highly protective against host injury. The lower CC_50_ for HFbs is not unexpected, as the literature suggests that NO susceptibility is cell type-dependent, with keratinocytes showing one of the highest levels of resistance to NO.^41^ In addition, when studying the effect of NO on skin cell growth, Krischel et al. discovered that keratinocyte proliferation and differentiation are regulated in a biphasic manner by different NO concentrations. In contrast, fibroblasts exhibited significant growth arrest even at the lowest concentrations of NO.^42^ Nevertheless, further toxicity testing, including in animal models, is necessary to advance the development of the SNO-MP as a therapeutic, and additional formulation modifications to the particle can be made to mitigate off-target effects.

In conclusion, our results underscore the potential of SNO-MP as a novel and effective treatment for onychomycosis. The ability to release large doses of NO, regardless of variations in temperature and incubation times, provides a foundation for formulation improvement and future therapeutic use. The large number and diversity of clinical strains tested further strengthen the findings. Although we found that *A. flavus* is resistant to the SNO-MP, we believe that overall onychomycosis treatment efficacy would not be significantly impaired, as this species is estimated to account for less than 2% of the total number of infections.^43^ The *ex vivo* nail infection model further corroborates the efficacy of SNO-MP in eradicating biofilms. It is worth highlighting that topical administration of SNO-MP is one of the major advantages of the proposed formulation, circumventing most of the pharmacological limitations associated with conventional oral antifungal therapy for onychomycosis, such as low bioavailability, lack of selectivity, and high toxicity.^11^ With additional, more comprehensive formulation studies and further development, these NO-releasing microparticles are a promising alternative to conventional treatments and appear poised to rapidly and effectively restore nail health. Additionally, the SNO-MP could be used in combination with traditional therapies, and future work will explore whether combinations are synergistic, additive, or antagonistic. Future studies with *in vitro* and additional *ex vivo* models will also accelerate the application of this promising technology to clinical practice.

## Supplementary Material



## Notes

Contain a table with information regarding all fungal strains used in this study and their respective donors; images of nail fragments with and without SNO-MP were also included as well as magnified images of the *ex vivo* nail infection with both *C. albicans* and *T. mentagrophytes* (PDF)
