# Supplementary material for: Nitric Oxide-Releasing Microparticles: A Novel Treatment for Onychomycosis
Source: Mol Pharm. 2025 Aug 21;22(9):5567–75. doi: 10.1021/acs.molpharmaceut.5c00613 (PMC12406251; doi:10.1021/acs.molpharmaceut.5c00613)
Supplement: Supplementary file 1 [file mp5c00613_si_001.pdf]

Nitric oxide-releasing microparticles: a novel treatment for onychomycosis.

Alessandro F. Valdez [1,2], Jhon Jhamilton Artunduaga Bonilla [1], Daniel Zamith-Miranda [2], Bruna Montalvão [1], Éva Veres [3], Sirida Youngchim [4], Clay Tucker [5], Andrew Draganski [5], Attila Gácser [3], Leonardo Nimrichter [1,6 §*], Joshua D. Nosanchuk [2 §]

1. Laboratório de Glicobiologia de Eucariotos, Departamento de Microbiologia Geral, Instituto de Microbiologia Paulo de Góes, Universidade Federal do Rio de Janeiro, Rio de Janeiro – RJ 21941-902, Brazil.
2. Albert Einstein College of Medicine, Departments of Medicine (Division of Infectious Diseases) and Microbiology and Immunology, Bronx – NY 10461, USA.
3. Department of Biotechnology and Microbiology, University of Szeged, Szeged 6726, Hungary
4. Department of Microbiology, Faculty of Medicine, Chiang Mai University, Chiang Mai 50200, Thailand
5. Zylö Therapeutics, Greenville, South Carolina 29615, USA
6. Rede Micologia RJ, Carlos Chagas Filho Foundation for Research Support of the State of Rio de Janeiro (FAPERJ), Rio de Janeiro – RJ 20020-000, Brazil.

§ LN and JDN are both the senior authors of this research.

* nimrichter@micro.ufrj.br

Abstract: Onychomycosis is one of the most prevalent fungal infections worldwide resulting in negative effects upon general quality of life. Management of the disease encounters a series of obstacles and antifungal treatment has a low success rate. We aimed to develop and evaluate a novel formulation of NO-releasing microparticle (SNO-MP) against some of the most common causative agents of onychomycosis: *Trichophyton mentagrophytes*, *Trichopyton rubrum*, *Candida albicans* and *Aspergillus flavus*. SNO-MP susceptibility testing was performed *in vitro* and MICs and MFCs stablished for multiple strains, SNO-MP was also tested *ex-vivo* in human nail fragments and biofilm formation, and biofilm disruption assessed with scanning electron microscopy and colony forming units counting. The particles demonstrated the ability to destroy biofilms formed by either *Trichophyton sp.* or *C. albicans*. SNO-MP was not effective against *A. flavus*, which appears to have a nitrosative resistance mechanism not yet elucidated. Cytotoxicity was assessed against human dermal fibroblasts and keratinocytes. SNO-MP demonstrated a manageable degree of cytotoxicity when tested against human dermal fibroblasts and keratinocytes. Our findings highlight that SNO-MP represent a potential potent alternative for the treatment of onychomycosis due to *Trichophyton* and *Candida*.

Keywords: Onychomycosis, *T. rubrum*, *T. mentagrophytes*, *C. albicans*, Microparticles, Nitric Oxide.

Fungal species	Strain	Origin
C. albicans	Sc 5314	ATCC
C. albicans	90028	ATCC
C. albicans	AV01-AV26	Dr. Erika Orner - Montefiore Medical Center (Bronx, NY - USA)
T. mentagrophytes	AV01, AV02	Dr. Erika Orner - Montefiore Medical Center (Bronx, NY - USA)
T. mentagrophytes	53062	Dr. Rodrigo Almeida Paes - Fiocruz (Rio De Janeiro , Brazil)
T. mentagrophytes	R1, FM1090	Dr. Carlos Taborda - University of São Paulo (São Paulo, Brazil)
T. mentagrophytes	DI21-100, DI23-103, DI23-104, DI23-107, DI23-108	Nathan O. Wiederhold – University of Texas Health Science Center at San Antonio (San Antonio, TX – USA)
T. mentagrophytes	PMN01, PMN02, PMN03	Dr. Paulo Murilo Neufeld – Federal University of Rio de Janeiro (Rio de Janeiro, RJ – Brazil)
T. rubrum	R1, FM1064	Dr. Carlos Taborda - University of São Paulo (São Paulo, Brazil)
T. rubrum	CFP900	Dr. Rodrigo Almeida Paes - Fiocruz (Rio De Janeiro , Brazil)
T. rubrum	AV01, BR1A	Dr. Erika Orner - Montefiore Medical Center (Bronx, NY - USA)
T. rubrum	DI23-110, DI23-112, DI23-113, DI23-114, DI23-116, DI23-118, DI23-119	Nathan O. Wiederhold – University of Texas Health Science Center at San Antonio (San Antonio, TX – USA)
T. rubrum	PMN01, PMN02	Dr. Paulo Murilo Neufeld – Federal University of Rio de Janeiro (Rio de Janeiro, RJ – Brazil)
A. flavus	AV01-AV03	Dr. Erika Orner - Montefiore Medical Center (Bronx, NY - USA)
A. flavus	SNS049, SNS091, SNS109, SNS110, SNS120, SNS144, TX1018, TX1021, TX1031, TX1041, TX1051, TX1084	Dr. Mark Weaver – US Department of Agriculture (Stoneville, MS – USA)

Supplementary Table 1. Fungal strains and donors.

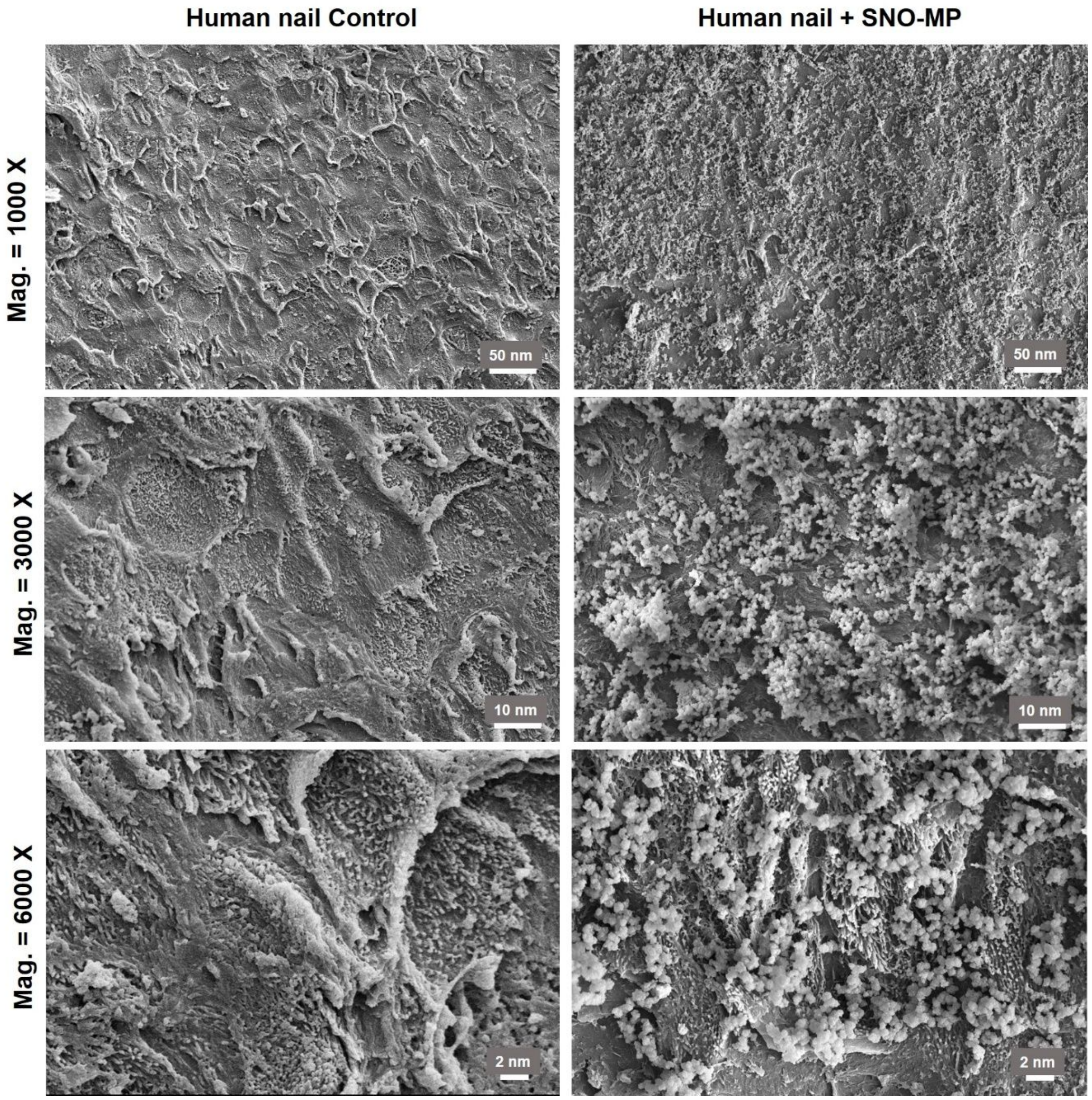

Supplementary Figure 1. Human nail fragments with and without SNO-MP at a 10 mg/ml concentration. Scale bars: at 1000X mag. = 50 nm, 3000X mag. = 10 nm, and 6000X mag. = 2nm.

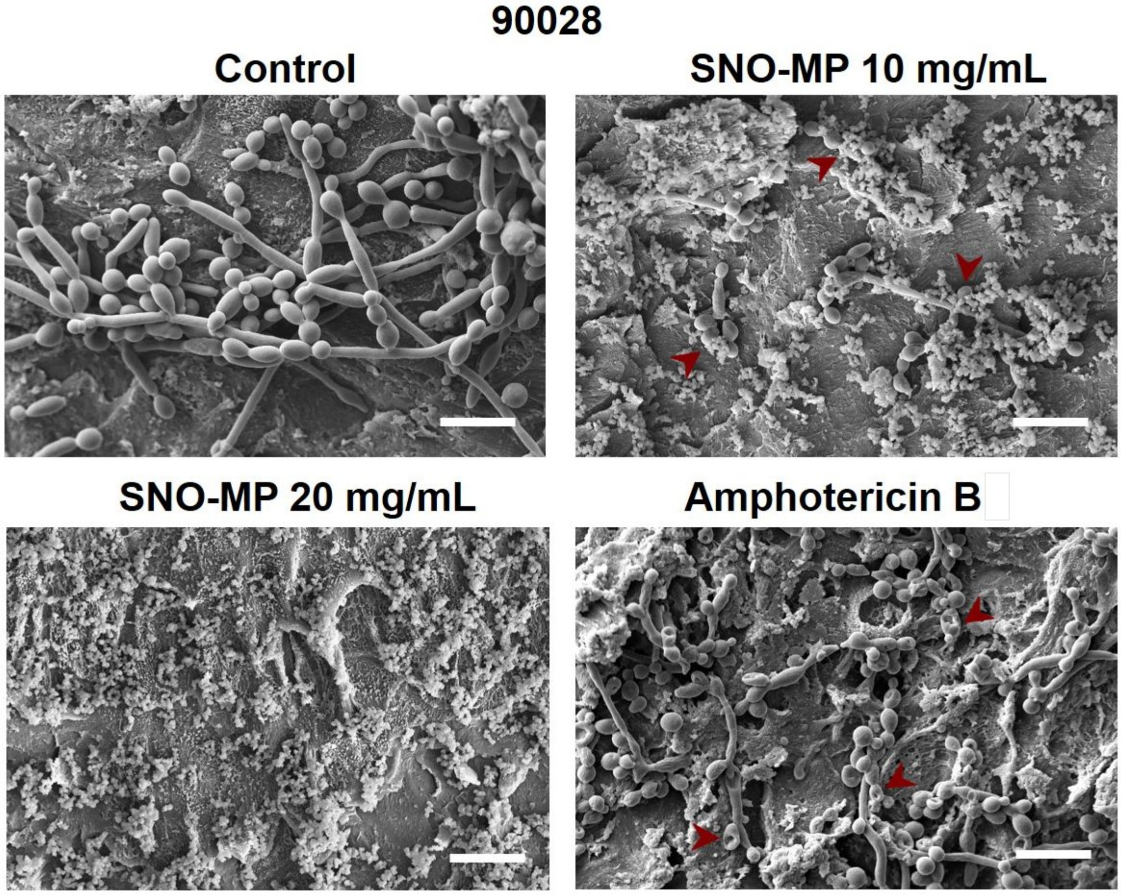

Supplementary Figure 2. Magnified image of *ex-vivo* efficacy of SNO-MP against *C. albicans* 90028. Nail fragments were incubated with *C. albicans* yeasts for 24 h at 37ºC. The fragments were then incubated with SNO-MP (10 and 20 mg/mL) or 1 µg/mL of AmB for the same period. Untreated infected fragments were used as controls. The micrographs show that the control group has yeast abundantly and uniformly occupying the entire nail surface. Treatment with SNO-MP at either concentration shows total destruction of fungal structures, with the few remaining yeasts showing signs of collapse and cellular stress (red arrows), similar to treatment with AmB. Scale bar = 15 nm. Representative images at a magnification of 3000x.

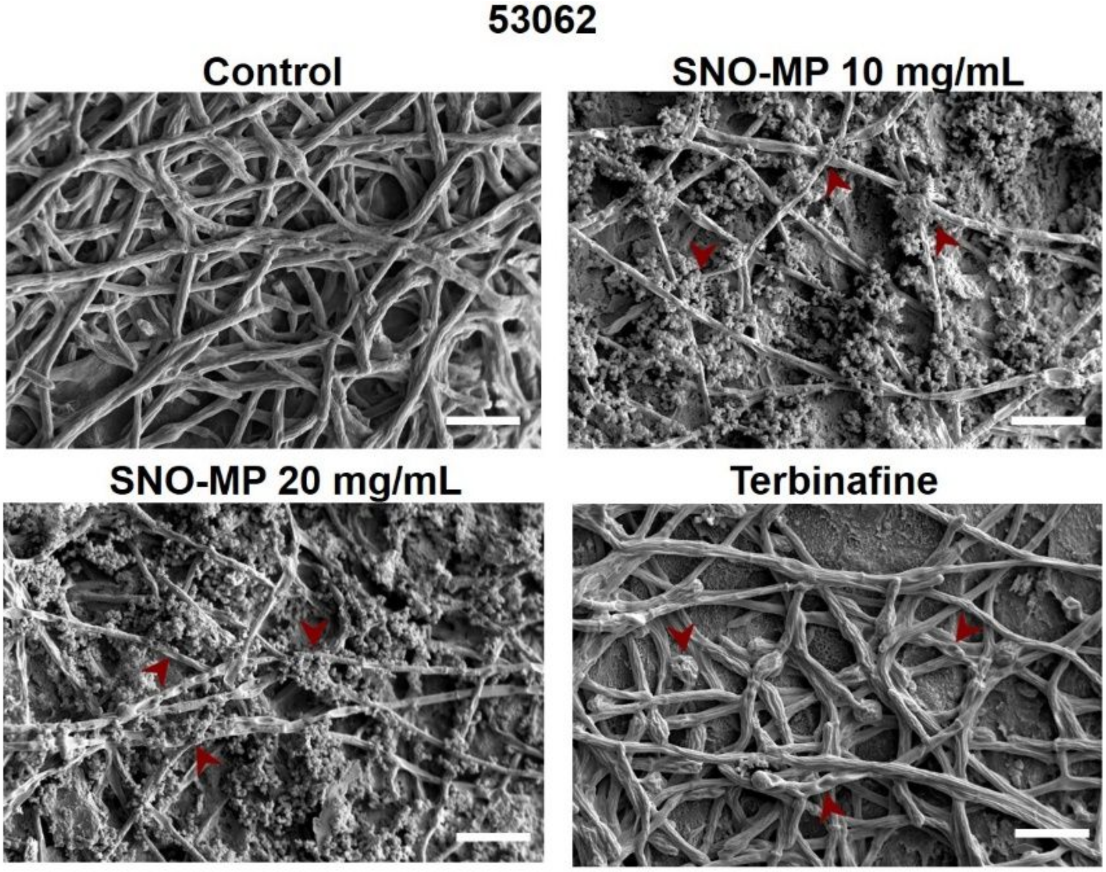

Supplementary Figure 3. Magnified image of ***ex vivo*** **efficacy of SNO-MP against *T. mentagrophytes 53062.*** Nail fragments were incubated with *Trichophyton* sp. for 72 h at 37ºC. The fragments were then incubated with SNO-MP (10 and 20 mg/mL) or 32 µg/mL of TRB for the same period. Untreated infected fragments were used as controls. The micrographs show that the control group has hyphae abundantly and uniformly occupying the entire nail surface. Treatment with SNO-MP at either concentration shows destruction of the fungal structures, with the few remaining hyphae showing signs of collapse and cellular stress (red arrows), similar to treatment with TRB. Scale bar = 15 nm. Representative images at a magnification of 3000x.
